# Micro-Fabricated RTD Based Sensor for Breathing Analysis and Monitoring

**DOI:** 10.3390/s21010318

**Published:** 2021-01-05

**Authors:** Bilel Neji, Ndricim Ferko, Raymond Ghandour, Abdullah S. Karar, Houssam Arbess

**Affiliations:** College of Engineering and Technology, American University of the Middle East, Kuwait; ndricim.ferko@aum.edu.kw (N.F.); raymond.ghandour@aum.edu.kw (R.G.); abdullah.karar@aum.edu.kw (A.S.K.); houssam.arbess@aum.edu.kw (H.A.)

**Keywords:** bridge circuits, respiratory rate, frequency measurement, micro-fabrication, thermal resistance, temperature sensors, wearable sensors

## Abstract

The design, micro-fabrication, and characterization of a resistance temperature detector (RTD) based micro sensor for minimally invasive breathing analysis and monitoring is presented. Experimental results demonstrate that the change in air temperature while inhaling and exhaling can be transduced into a time varying electrical signal, which is subsequently used to determine the breathing frequency (respiratory rate). The RTD is placed into a Wheatstone bridge to simultaneously reduce the sensor’s output noise and improve overall system accuracy. The proposed design could potentially aid health care providers in the determination of respiratory rates, which is of critical importance during the current COVID-19 pandemic.

## 1. Introduction

Respiratory diseases are a major cause of death worldwide. The current corona virus disease (COVID-19) pandemic is a prime example of severe acute respiratory distress syndrome (SARS) [1]. In order to limit the spread of the corona virus, early diagnoses is of critical importance. Recent studies found that abnormal respiratory symptoms are important factors in the diagnosis of the disease. In general, COVID-19 patients exhibit a wide range of respiratory symptoms such as shortness of breath, atypical and irregular breathing. These symptoms can be considered as an early indicators of the disease progression [2,3,4]. Recently, sensors were developed to target the early detection of respiratory distress symptoms, such as a wearable stethoscope for respiratory health monitoring [5,6,7], infrared sensors for temperature monitoring [8], magnetic field proximity sensor [9], cell-based biosensor [10] and a remote health monitoring based signal processing techniques [11].

Measuring the breathing frequency $f_{b}$ (known clinically as the respiratory rate) is not only an informative human vital signs [12], but also offers a metric for the early detection and diagnoses of SARS during the current COVID-19 pandemic [13]. Contrarily, some serious illnesses (e.g., sleep apnea) can also be monitored by detecting variations in breathing rate or abnormal respiratory rate [14]. In order to measure the respiration, one needs to detect it. Resistance temperature detector (RTD) devices has to be developed and used to detect the breathing frequency and temperature. This is due to its linearity, measurement repeatability and stability [15,16].

Several techniques have been used to measure breathing frequency. These techniques use either contact or contact-less methods [17,18]. Contact-less methods are more complicated and require external devices, which can be expensive and impractical for a variety of applications. This work focuses on a minimaly invasive contact-based sensor to ensure accuracy and reliability.

The paper is organized as follows. Section 1, introduces the scope and importance of the research. Section 2, provides a brief overview of the different methods used for breathing analysis and monitoring. A few commercial and research devices are discussed. Section 3, is dedicated to the proposed sensor design where simulation results are presented and the final design is introduced. Section 4, elaborates the physical design of the sensor and its optimization. Section 5, describes the system implementation and test results. Section 6, is dedicated for conclusions.

## 2. Breathing Analysis Methods Overview

Breathing is a vital sign for human beings, therefore analyzing it is a crucial task. Various technologies are available for measuring $f_{b}$ such as contact or contact-less. For the contact-based techniques, the sensor must be in contact with the subject’s body, whereas that is not the case for the contact-less based techniques. Based on the physical and/or chemical principles applied for the operation of the sensor, there are different types of techniques used, such as flow, acoustic, temperature, humidity, CO^2^, light intensity, strain and movement measurements. A survey of the most relevant methods are outlined in the following subsections.

### 2.1. Flow Measurement Techniques

The breathing process includes inhaling and exhaling. Different sensors can be used to measure the volume or velocity of the breathing process. Among these sensors are differential flowmeters (DFs) and hot wire anemometers (HWAs). DFs consists of a pneumatic resistance positioned inside the pipe in which the gas flows. The resistance transduces the gas flowrate within a pressure drop with a well-known relationship called Hagen-Poiseuille law. Unfortunately, in order to work properly these sensors need a differential pressure sensor as a secondary element to measure the pressure drop in real-time. There are two main types of DFs used for measuring $f_{b}$—Pneumotachographs and Orifice meters. In the case of Pneumotachographs, the Hagen-Poiseuille law expresses a linear relationship between the input and the output. One concern regarding the Pneumotachographs is related to the influence of the gas composition and the temperature on their response. Such factors directly affect the value of dynamic gas viscosity, which is part of the input/output relation. Orifice meters can be subdivided into fixed orifice meters, where the resistance is an orifice plate, and variable orifice meters, where the plate increases its passage area as the flowrate increases [19]. In both cases, the input output relationship is expressed as a non-linear relation. Furthermore, the need to collect the entire inhaled and exhaled airflow via a collector causes a problem due additional resistance, which may not be tolerated in most applications.

Hotwire Anemometers consist of one or more heated wires exchanging heat with the airflow. The equilibrium temperature of the wire is expressed in [20]. King’s law is used to express the relationship between the coefficient of heat transfer and the velocity of gas that hits the hot wire [21]. HWAs in general exhibit a nonlinear response. Another concern for HWAs is its fragility due to the small size of the wire. Moreover, the typical configuration with a single wire can detect either inhaling or exhaling at a time; hence, more complex configurations are needed, as described in Reference [22].

### 2.2. Temperature Measurement Techniques

During the breathing process, the exhaled air is warmer than the inhaled air. The difference in the temperature between exhaling and inhaling can reach up to 15 degree Celsius as demonstrated in Reference [23]. By using this principle, different sensors can be used to measure the $f_{b}$. The most common sensors are thermistors and thermocouples, pyroelectric, and Fiber Bragg Grating sensors (FBG). A thermistor is a type of resistor that with temperature variation changes its value. There are two types of thermistors: negative thermistor, whose resistance drops when the environmental temperature increases, and positive thermistor whose resistance increases when the environment temperature increases. The relation between temperature and resistance is expressed by the non-linear Steinhart-Hart equation. This limits the temperature span of thermistors to about 100 degree Celsius, which is not considered a disadvantage as the temperature range of interest is between 10–40 degree Celsius. The size of thermistors is quite small and they are relatively cheap, which facilitates their application as reference instruments in many studies. Thermocouples, on the other hand, use the Seebeck effect as a principle to explain the relation between voltage and temperature [24,25]. When two different conductors are connected, the electromotive force that occurs is proportional to the temperature of the free ends and the temperature of the junction between them. The authors of Reference [26] stated that some material combinations to form a thermocouple are better than the others. The accuracy of thermocouples is similar to that of thermistors, however, their higher cost places them at a disadvantage. Pyroelectric sensors make use of the random motion caused by thermal agitation when the sensor is heated. This event generates a charge caused by the reduction in the transducers average polarization. The output current is proportional to the rate of temperature change [27] and is measured by the Stefan-Boltzmann law. The FBG is one of the most common wavelength-selective fiber components and is utilized in fiber resonators operating at the so called Bragg Wavelength $\lambda_{b}$. FBGs are an excellent tool to measure temperature variations, since the sensitivity to temperature is encoded directly in the $\lambda_{b}$ [28].

### 2.3. Humidity Measurement Techniques

The inhaled air has a relative humidity (RH) of the environment, which is approximately between 40–80%, while the exhaled air is saturated by vapor and has a 100% RH [29]. This RH difference between inhaled and exhaled air can be used to sense and measure the $f_{b}$. The principle is based on the sensitivity of a predefined electric parameter to RH. The most common are the capacitive and resistive based sensors. The capacitive sensors are widely used and represent more than 75% of the market share [30]. The principle is based on capacitance change with the dielectric properties of the material between the two electrodes of the capacitor [31]. The dielectric (i.e., polymer or ceramic material) deposited between the parallel electrodes will either absorb the water vapor when the RH increases or release it when the RH decreases. Since the capacitance depends on the dielectric properties, the changes can be used to sense the RH variations. Similarly, for resistive sensors, the change in RH affects the value of the resistance. For both cases, a specific circuit is used to express the relation between the input and the output. Generally humidity measurement techniques generally are only convenient for indoor usage as they are easily influenced by changes in the environmental factors. Their response time can vary from few milliseconds to a few seconds depending on the used materials.

### 2.4. $CO_{2}$ Measurement Techniques

During the inhaling, the $CO_{2}$ percentage is around 0.04% (<300 parts per million, ppm) and during exhalation around 6% (≈60,000 ppm) [32]. In such context, the detection of $CO_{2}$ can be used to measure the $f_{b}$. Infrared sensors are a common method used to detect the presence of $CO_{2}$ where the main components are an infrared source, a light tube, an interference filter and an infrared sensor. The gas is pumped into the light tube where the absorption of the characteristic wavelength of light is measured. A non-dispersive infrared sensor (NDIR) is used and the principle is explained in Reference [33]. The drawback of gas measurement techniques is the need for many components, which makes their cost relatively high and calibration difficult. Another technique used is the fiber-optic sensor where a $CO_{2}$ sensitive material is positioned at the end of the fiber transferring the light. Similarly, to the infrared sensor, the fiber optic sensor has a high cost and is sensitive to environmental factors.

### 2.5. Strain Measurement Techniques

During the inhaling and exhaling process air enters the lungs causing the chest to expand up to 7 cm [34], which could be used in measuring the $f_{b}$. This techniques transform the cyclic expansion and contraction of the chest into a signal that is used to extract the breathing frequency. Among the different strain sensors, the piezo resistive strain sensor is the most common type used in detecting the externally applied strain, which modifies the geometry of a piezo resistive sensing element [17]. Such sensor exhibits poor durability which leads to a lower performance and is easily affected by other motions not related to breathing such as walking and speaking [35].

## 3. Proposed Sensor Design Optimization & Simulation

**Figure 1:**
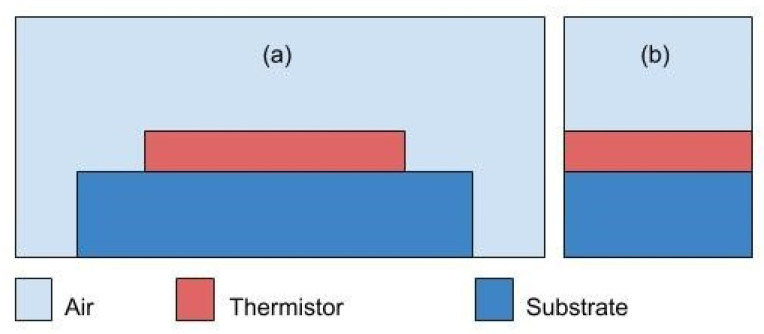
General sensor design; (**a**) x-y plane; (**b**) z-y plane.

In this paper, we focus on temperature measurement technique, more precisely thermistors. Figure 1 shows the general sensor design which consists of three different layers. The substrate is the base film where the thermistor layer will be deposited. The thermistor layer is the sensing component of the design, which is composed of a material sensitive to temperature. Figure 1a, illustrates the x-y plane, while Figure 1b, illustrates the z-y plane corresponding to the cross-sectional cut of the design.

### 3.1. Sensor Design Optimization

**Figure 2:**
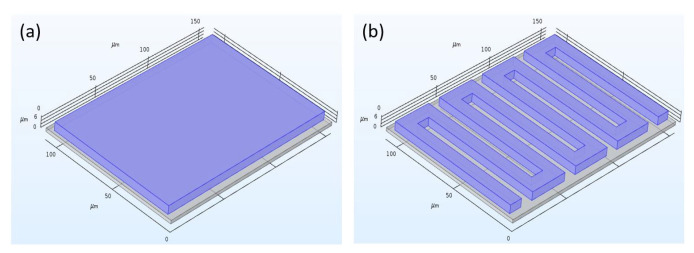
Thermistor shapes (**a**) Rectangular; (**b**) Serpentine.

The proposed design uses the thermistor principle to measure the change in air temperature. In order to accomplish optimal results and performance, the proposed design was verified for different materials, different thermistor thicknesses, and shapes. The first layer represents a very thin substrate made of glass material. Glass has a very low thermal conductivity, which is 100 times smaller than that of silicon. Low thermal conductivity is essential for preserving unwanted heat loss to the substrate and the surroundings of the sensor. The second layer is the thermistor which is the most crucial and important layer of the design. In our designing steps, we have considered three main variables, thermistor shape, size and material. At first, we tested two different shapes as shown in Figure 2, where (a) is a rectangular shape and (b), is a serpentine shape.

**Figure 3:**
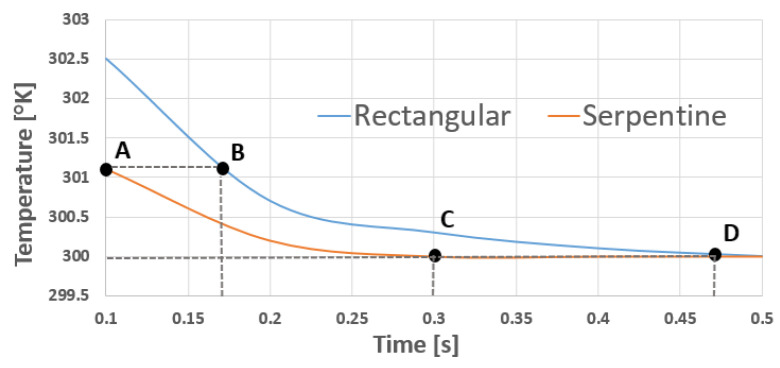
Temperature vs. Time for different shapes: rectangular and serpentine.

As observed in Figure 3, points A and B correspond to different response times for different shapes at 301.1 degrees kelvin, and points C and D correspond to different response times for different shapes at 300 degrees kelvin, which indicates that the performance of the serpentine shape is better than the rectangular shape. The advantage of reshaping of the rectangular resistor into a serpentine shape, while maintaining the same overall dimensions, is twofold. Firstly, increasing the sensor sensitivity through increasing the resistance. Secondly, the contact area with the inhaled and exhaled air is increased through a larger surface area, allowing for a wider measuring apparatus. This validates the statement in Reference [36] where a serpentine resistor is capable of having a resistance value two orders of magnitude higher than a rectangular resistor.

**Figure 4:**
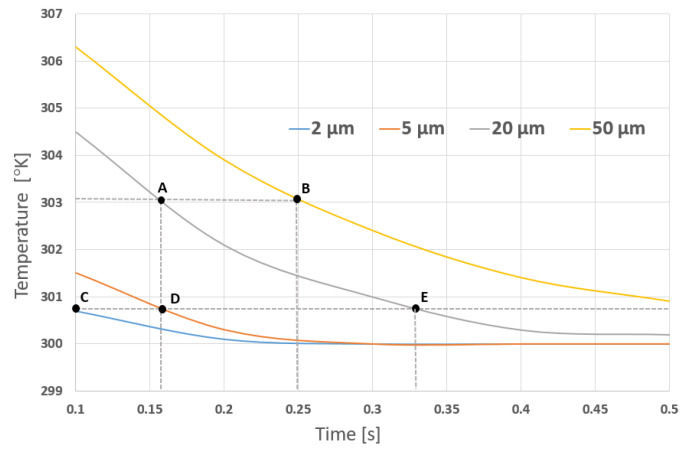
Temperature vs. Time for different thicknesses.

After deciding the shape, the design is verified at different thicknesses. Figure 4, shows the simulations for different thermistor thicknesses. Points A and B show the response time of the sensor using different thicknesses at 303.1 degrees kelvin. Points C, D and E show the response time of different thicknesses at 301 degrees kelvin. It can be observed that, the heat transfer to the thermistor decreases as the thickness increases. As a result, we chose a thickness of 5 μm. Lastly, after we have selected the best performing shape and thickness, we simulated the design for different materials. The following simulation has a fixed shape and thickness where the changing variable is the material.

**Figure 5:**
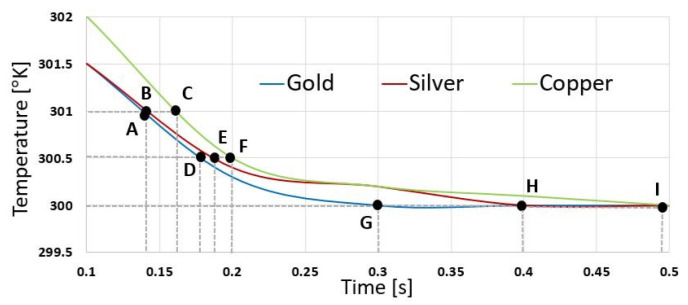
Temperature vs. Time for different materials.

As observed in Figure 5 points A, B, and C show the response time for each of the materials at 301 degrees kelvin. Points D, E and F show the response time for each material at 300.5 degrees kelvin, and points G, H and I show the response time for each material at 300.5 degrees kelvin. We can observe that the best response is from gold.

**Figure 6:**
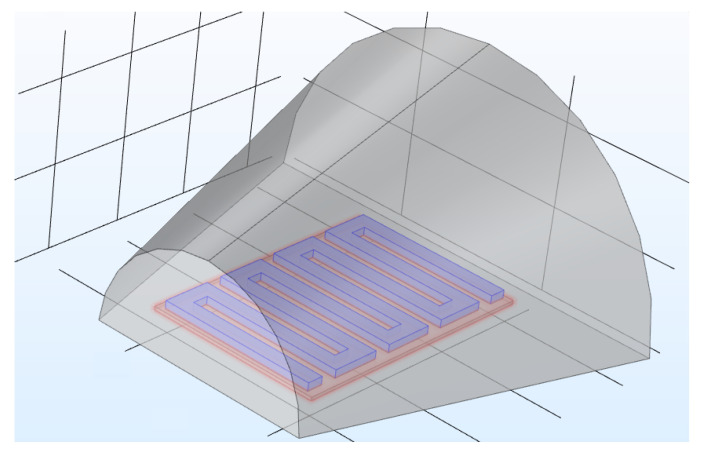
Final sensor design.

The last layer represents the air surrounding the sensor and the substrate. We have tried to mimic the inside of the nose shape, which has a cylindrical shape as shown in Figure 6. The red region represents the glass substrate; the blue region represents the serpentine shape thermistor made of gold with a thickness of 5 μm.; the outer region in grey represents the air.

### 3.2. Final Proposed Design

**Figure 7:**
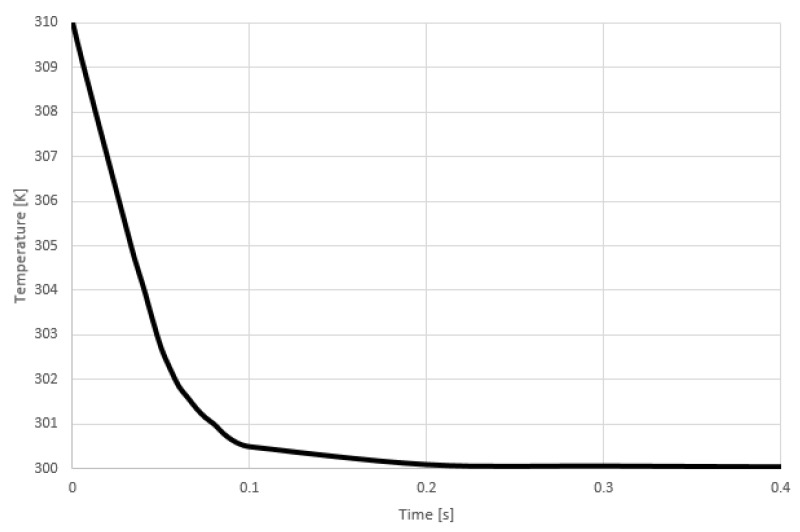
Temperature vs. Time during inhaling process.

The simulation results demonstrate that the best design for the RTD sensor is the serpentine shape and the most convenient material is gold. The breathing process consists of inhaling and exhaling. When inhaling occurs, the temperature of the air entering the nose is around room temperature, which is considered to be 300 degrees kelvin. On the other hand, the temperature of the human body is around 310 K, which is 37 degree Celsius. Figure 7, shows the inhaling process where the air flows from the nasal entrance towards the inner part of the nose.

**Figure 8:**
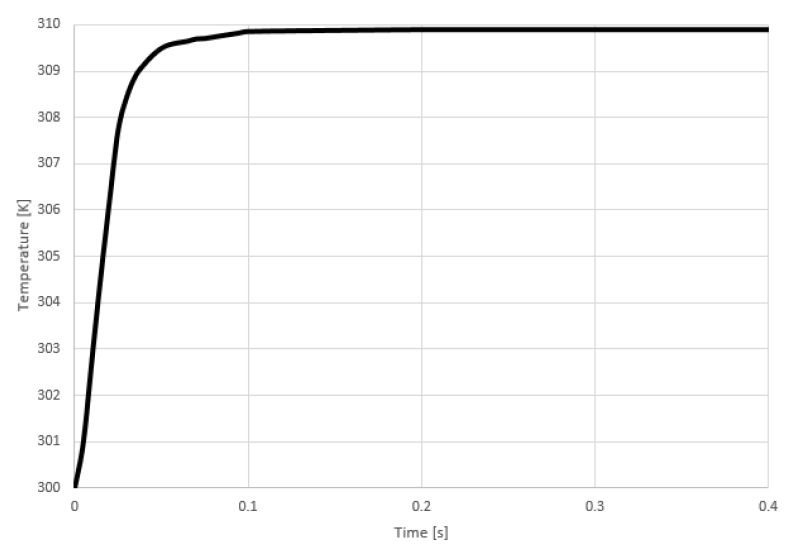
Temperature vs. Time during exhaling process.

Figure 8, shows the exhaling process where the air flows from the inner part of the nose towards the nasal entrance. It can be observed that inhaling or exhaling results in a temperature change of around 10 degrees kelvin. It was observed from the simulation results, that the signal stabilizes after 0.2 s, which is considered as the response time of the system.

The RTD sensor will be mounted inside the patient’s nose and will be connected to a Wheatstone bridge circuit which will be discussed in the following section.

## 4. Sensor Physical Design & Optimization

In this section, the sensor micro-fabrication process is presented. In addition, an initial physical design proposal with the related simulation results are illustrated. Finally, enhanced sensor design and its simulation and results are presented.

### 4.1. Fabrication Process

**Figure 9:**
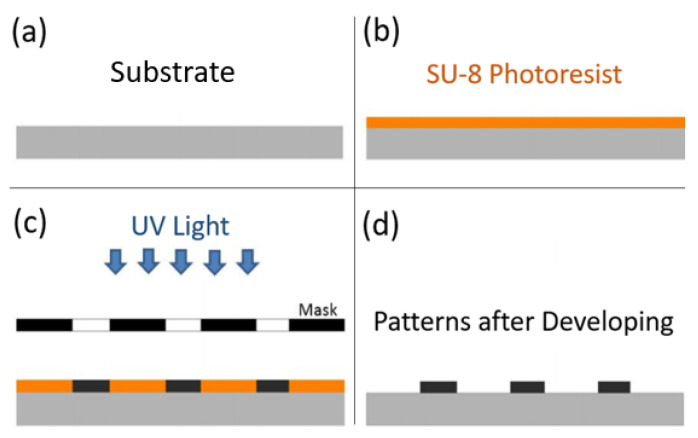
General process for single-depth photo-lithography: (**a**) Substrate preparation; (**b**) Spin coating; (**c**) UV exposure; (**d**) Development.

The micro sensor has been fabricated in a class 1000 clean room. Photo-lithography is employed to create the sensor’s patterns. The general procedures used in single depth photo-lithography is presented in Figure 9.

A negative photo-resist is placed on the substrate and then rotated at 4500 rpm for 30 s. Then, the substrate is placed on a hot plate for pre-baking. The purpose of the pre-bake is to eliminate excess coating solvent and harden the photo-resist. The patterns are then transferred from the mask to the photo-resist using UV exposure. Lastly, the substrate is dipped into a developer for about 4 to 5 min. The part exposed to the UV light remains while the other part gets dissolved [37].

**Figure 10:**
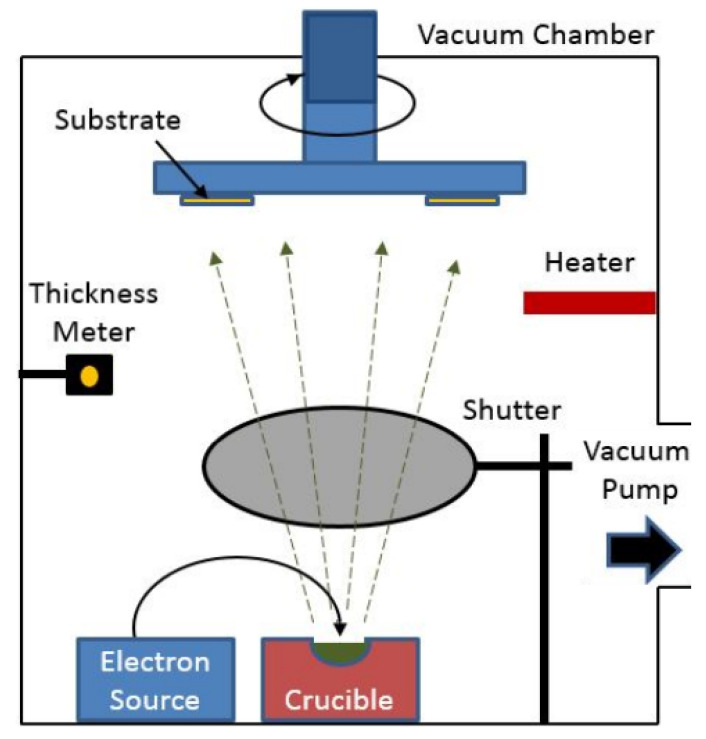
Simplified Electron beam evaporation system diagram.

Electron beam evaporation machine is used for metal deposition, including silver, gold, and aluminum. The technique is based on heat generation from a high-energy electron beam bombardment on the material to be deposited. Therefore, emitted electrons from the electron source are accelerated towards an anode by a high difference of potential in the order of kilo-volts. The crucible itself or a near perforated disc can act as the anode. A magnetic field is used to bend the electron trajectory. Figure 10, presents a simplified illustration of the used evaporation system.

### 4.2. Inhaling Sensor Proposal

The breathing frequency sensor is mainly composed of a resistance temperature sensor and conductive lines also called connections to pads. The goal of the initially proposed designs is to demonstrate the operation of the device and optimize its function. In the following, three design revisions of the frequency-breathing sensor are presented—revision 0.1, revision 0.2, and revision 0.3.

#### 4.2.1. Design Revision 0.1

**Figure 11:**
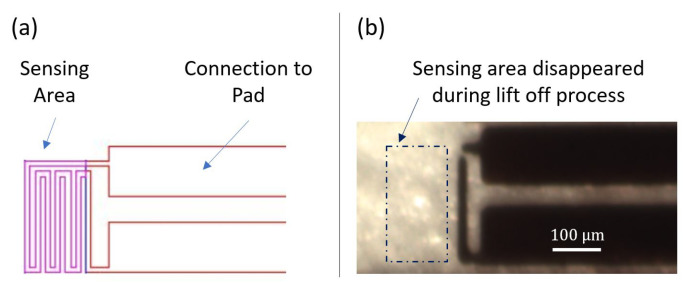
(**a**) Design revision 0.1; (**b**) Micro-fabricated sensor.

The first revision of the sensor design is shown in Figure 11a. The sensing element, mainly the resistor, is 10 μm wide and 1600 μm long. The overall area of the sensing element is 150 μm × 250 μm. The spacing between the two connecting line patterns is 10 μm. The width of the connection to pads is 110 μm. Aluminum has been used to fabricate the sensor, including the sensing element and the connections to pads. The sensor patterns were deposited on a glass substrate. Figure 11b, presents a microscopic picture of the fabricated sensor. After metal deposition and lithography, most of the patterns with very narrow width disappeared during the lift off process, including the serpentine sensing shape. This can be the consequence of either a long time lifting process or exceeding the maximum resolution of the mask aligner, which is theoretically equal to 10 μm. Therefore, revision 0.2 of the sensor design is proposed to eliminate the issues faced in design revision 0.1.

#### 4.2.2. Design Revision 0.2

**Figure 12:**
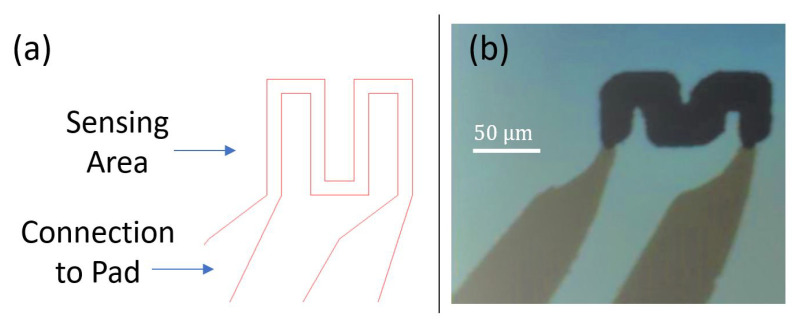
(**a**) Design revision 0.2 (**b**) Micro-fabricated sensor.

Revision 0.2 of the sensor design includes few improvements. First, the width of the sensing element has been increased to 20 μm to avoid going below the practical maximum resolution. Second, the spacing between patterns has been raised to 40 μm. Lastly, the connection to each pad has been slightly changed in a way that its width gradually increases starting from the sensing element until it reaches the pads. This will help avoid the sudden change in resistance at the junction between the sensing pattern and the connection to each pad. Revision 0.2 of the sensor design is illustrated in Figure 12a. The sensing pattern is 20 μm wide and 760 μm long. The overall area of the sensing element is 200 μm × 160 μm. In this revision, copper was used to fabricate the connections to pads, and aluminum was used to make the sensing element. The idea is to test the performance of using two different materials for two connected structures. Similarly, to revision 0.1, all patterns were deposited on a glass substrate. Figure 12b presents a microscopic picture of the fabricated sensor.

It can be concluded that most of the issues faced in revision 0.1 were resolved. However, we noticed that the junction at the connection between the sensing element and the connections to pads has a huge resistance. This issue is due to the formation of a layer of isolating oxide at the junction between the two metal layers during the time between the first and second depositions. This could be due to aluminum exposure to air before the start of copper deposition. Therefore, revision 0.3 of the sensor design is proposed to eliminate the issues raised in revision 0.2.

#### 4.2.3. Design Revision 0.3

**Figure 13:**
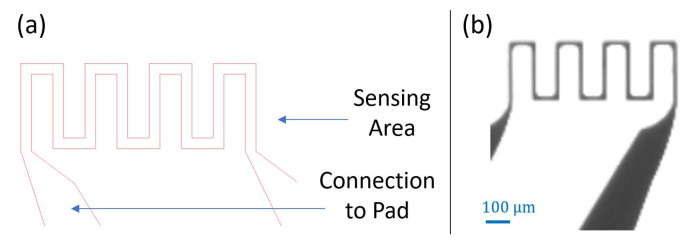
(**a**) Design revision 0.3 (**b**) Micro-fabricated sensor.

Few improvements have been implemented in revision 0.3. To start with, the length of the sensor has been augmented to increase the sensing contact surface and get better readings. Second, the same metal has been used to deposit both the sensing area and the connections to pads. Sensor design revision 0.3 is shown in Figure 13a. The width of the sensing pattern is 20 μm, and the length is 1.56 mm. The overall area of the sensing element is 440 μm × 160 μm. The sensor patterns are deposited on a glass substrate. Figure 13b presents a microscopic picture of the fabricated revision 0.3 sensors. It can be noticed that the patterns are clear and well deposited.

#### 4.2.4. Simulation Results

**Figure 14:**
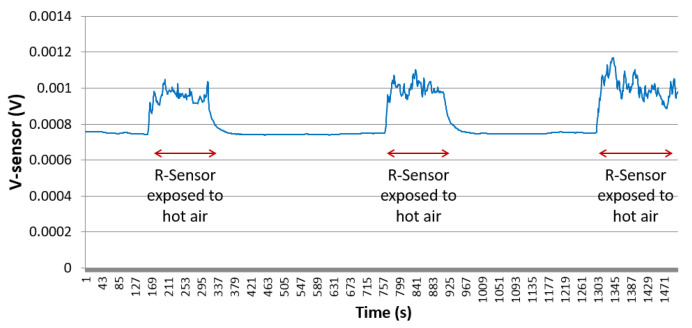
Sensor output voltage over time, as function of air temperature surrounding the sensing element.

In order to demonstrate the operation of sensor revision 0.3, the voltage across the sensing element is measured at different applied heat values at the proximity area of the sensing element. The measured voltage is proportional to the sensing element resistance, which is a function of its surrounding temperature. Figure 14 presents the sensor’s output voltage overtime at different conditions: readings at room temperature, then readings while exposed to around 340 degrees kelvin hot air.

The simulation results clearly validate the operation of the micro fabricated sensor. A variation of the sensor’s output voltage of about 0.2 mV can be observed when the temperature around the sensing element increases by around 30 degrees Kelvin. Even though the applied temperature around the sensor is constant and stable, it can be noticed that the sensor’s output voltage reading is not stable enough to be used to differentiate between inhaling and exhaling scenarios. At temperature, around 340 degrees Kelvin, the sensor’s output voltage is fluctuating between 0.85 mV and 1.05 mV. This results in a reading error of about 0.2 mV for a temperature variation of 30 degrees kelvin. Therefore, sensor design revision 0.3 is not suitable for breathing frequency determination. In the following subsection, enhanced sensor design is proposed.

### 4.3. Enhanced Sensor Proposal

**Figure 15:**
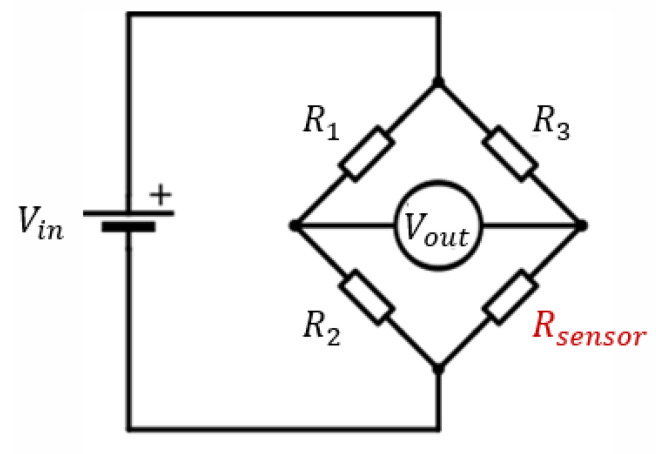
Wheatstone bridge circuit schematic.

In order to solve the issue faced in design revision 0.3, a Wheatstone bridge is used to accurately measure the resistance change when the temperature around the sensor changes. A Wheatstone bridge can be used to convert a small sensor’s resistance change, due to a surrounding small temperature variation, into an accurate output voltage across the sensing element [38,39]. As shown in Figure 15, one pair of the bridge pads is used to apply a DC voltage, in our case $V_{in}$ = 0.5 V, and the other pair is used to measure the output voltage. The relationship between $V_{out}$, $V_{in}$ and $R_{sensor}$ are given in Equation (1).

(1) $$V_{out}=\left(\frac{R_{sensor}}{R_{3}+R_{sensor}}-\frac{R_{2}}{R_{1}+R_{2}}\right)\times V_{in}.$$

#### 4.3.1. Sensor Design Revision 1.0

**Figure 16:**
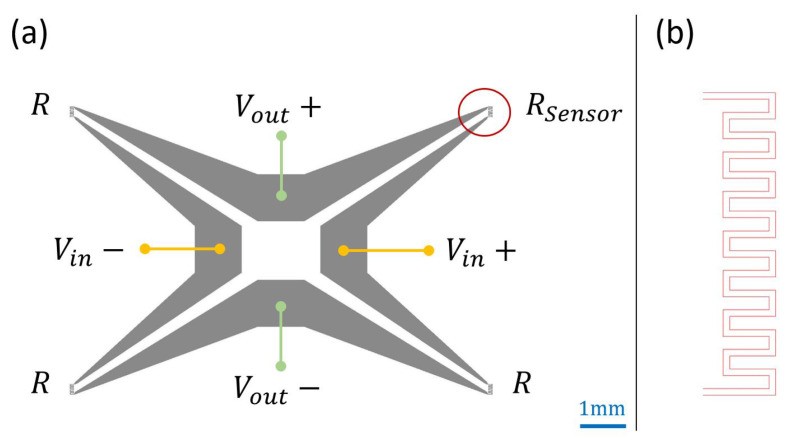
(**a**) Sensor design revision 1.0 with Wheatstone bridge (**b**) Sensing element design.

The enhanced revision of the sensor design is composed of four identical resistors making a closed-loop as shown in Figure 16a. The resistors’ design is very similar to the sensing element in revision 0.3, except that the length has been doubled to increase its contact surface (about 3 mm long). Three sensors, with resistance R, have been covered to be isolated and not affected by any heat applied at the sensing element proximity. All patterns have been deposited using the same material to avoid high resistances at the junctions (issue faced in design revision 0.2).

**Figure 17:**
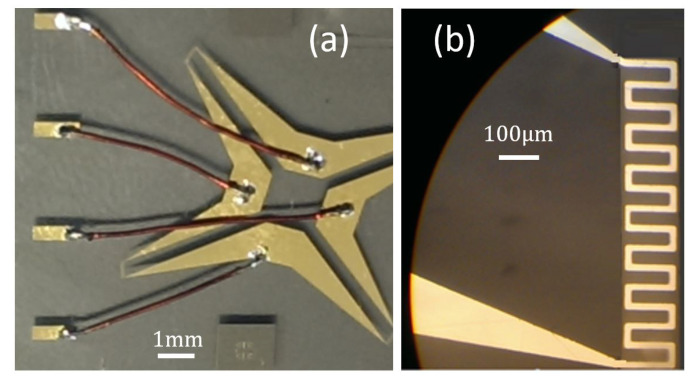
(**a**) Micro fabricated gold sensor, design revision 1.0 (**b**) Close up of the sensing element.

Several materials have been used to fabricate the device, including aluminum and gold. The picture in Figure 17a shows a fabricated sensor using gold. All sensor patterns were deposited on a glass substrate. Thin copper wires have been used to connect the bridge to Vin and Vout. A close up of the sensing element is presented in Figure 17b.

#### 4.3.2. Simulation and Results

**Figure 18:**
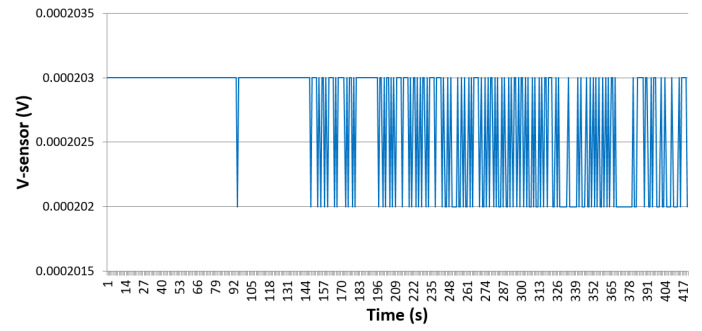
$V_{out}$ vs. time at a constant applied heat surrounding the sensing element.

Several devices, with design revision 1.0, have been micro fabricated using gold to validate the different proposed enhancements. The bridge output voltage $V_{out}$ has been measured while applying a constant heat at the surrounding of the sensing element (Figure 18). It can be observed that the sensor reading is stable with a maximum error of 1 μV. Compared to the results obtained for sensor revision 0.3, with a reading error of 0.2 mV, the usage of Wheatstone bridge in design revision 1.0 improved the reading accuracy by a factor of 200 times.

**Figure 19:**
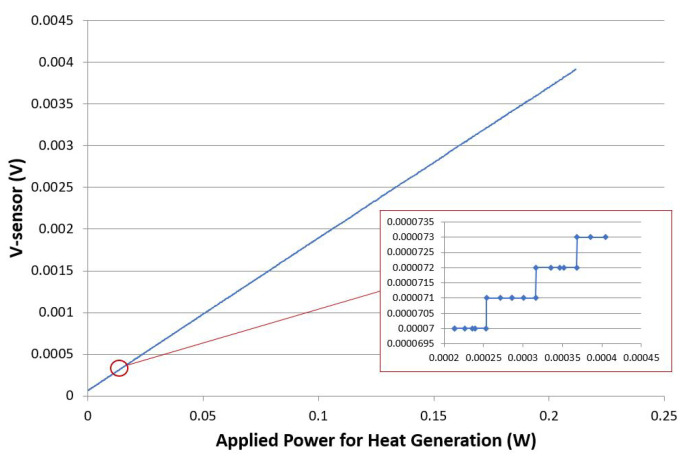
$V_{out}$ as function of the applied heat around the sensing element.

$V_{out}$ as a function of the applied heat around the sensing element is presented in Figure 19. An increment of 50 μW. at a heating element located in the sensor’s surrounding corresponds to an increment of 1 μV. at the bridge output voltage. This increment is illustrated in the reading zoom-in presented in Figure 19.

The sensitivity of the micro fabricated sensor has been determined by calculating the ratio between the sensor’s output voltage and the applied power to the heating element surrounding the sensor, which corresponds to the slope of the curve in Figure 19. Therefore, it could be concluded that the sensor’s sensitivity is in the order of 20·$10^{-1}$$A^{-1}$. Furthermore, It could be deduced from the results in Figure 19. that the sensor has a linearity of 0.998 (from 0 W to 0.2 W applied power to the heating element surrounding the sensor). The obtained results for the enhanced sensor design demonstrate that the device can be comfortably used to determine the breathing frequency by measuring the inhaling and exhaling breathing temperatures over time.

## 5. System Implementation & Results

### 5.1. Experiment Setup

**Figure 20:**
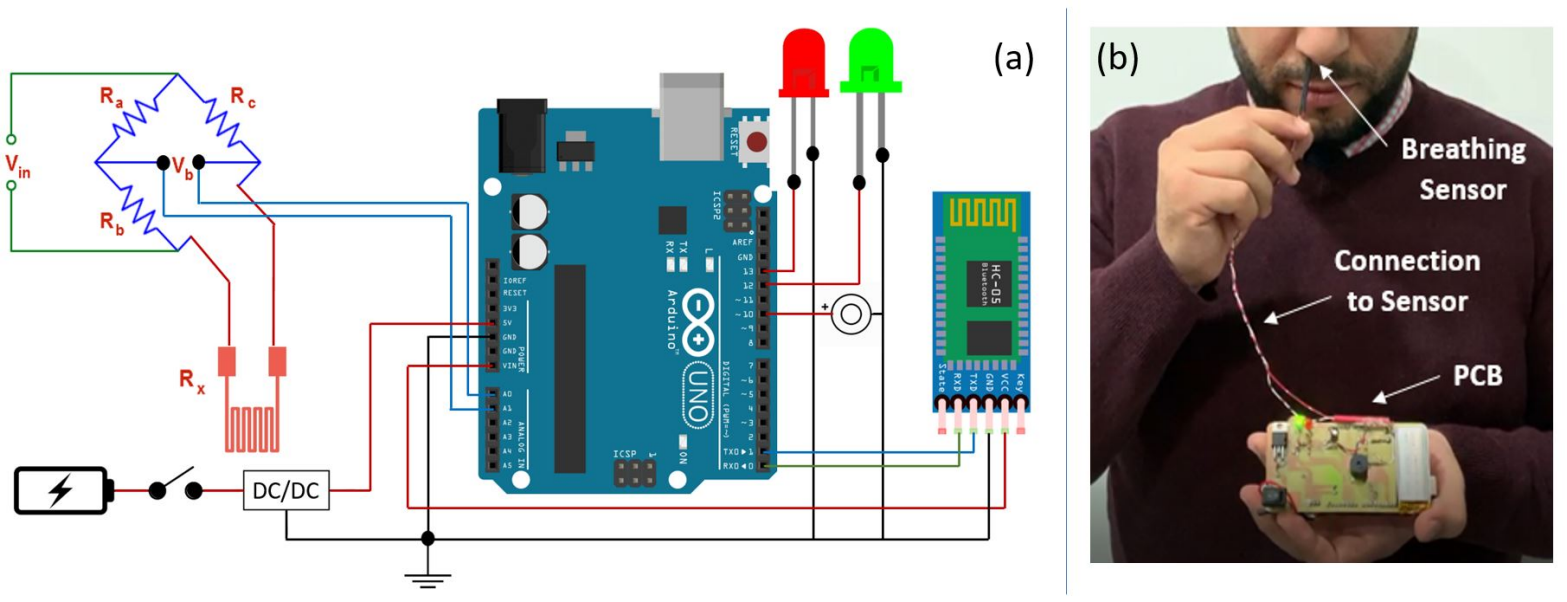
(**a**) System block diagram (**b**) Experimental setup.

Several types of equipment are used to determine the performance of the RTD based sensor. The block diagram of the system and the measurement setup is shown in Figure 20a,b, respectively. A printed circuit board (PCB) was constructed and several electronic components such as LED, Buzzer, Battery, Arduino UNO, Bluetooth module, Resistors, Capacitors, RTD sensor, Voltage regulator and Switch were connected. The sensor is placed inside the patient’s nose, which is able to read the breathing frequency. The Arduino board reads the signal coming from the RTD sensor and sends the data via a Bluetooth module to a PC for further analysis.

All subjects gave their informed consent for inclusion before they participated in the study. The study was conducted in accordance with the Declaration of Helsinki, and the protocol was approved by the Ethics Committee of (SP190402-02).

### 5.2. Experiment Results

**Figure 21:**
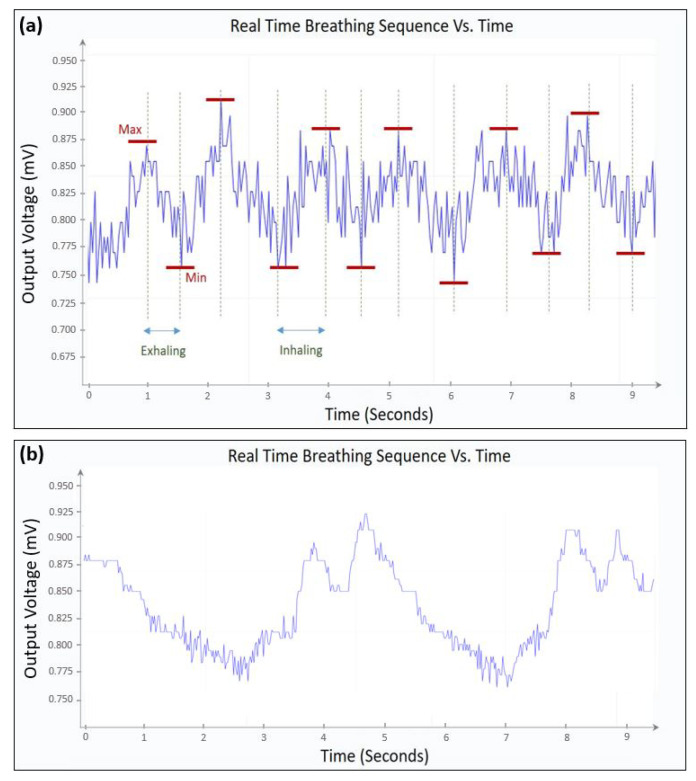
(**a**) Measured output voltage vs. time for a healthy patient. (**b**) Measured output voltage vs. time for a patient with sleep apnea.

Figure 21a,b, represent the sensor’s output voltage, which corresponds to inhaled/exhaled air temperature, as function of time. A normal pattern corresponds to the sensor’s response in the case of a patient breathing normally. On the other hand, an abnormal pattern corresponds to the sensor’s response in the case of a patient breathing fast or slow. What distinguishes healthy patient from an unhealthy patient is the breathing frequency at different conditions. A healthy patient usually has a breathing frequency above 0.17 Hz and that could change depending on different stressors, as mentioned by the authors in Reference [40]. Respiratory rates below this value should be reported to a doctor. s shown in Figure 20a, the system has two LEDs to indicate whether the test subject has a normal or an abnormal breathing frequency. A green LED refers to a normal breathing pattern, whereas a red LED and a buzzer indicate an abnormal breathing pattern.

In Figure 21a the decrease of the output voltage from “max 1” to “min 1”, corresponds to a decrease in the sensing resistance, which indicates that the temperature is decreasing, demonstrating an inhaling pattern. On the other hand (assuming that the room temperature is lower than the body temperature), the increase of the output voltage corresponds to an increase in the sensing resistance, which indicates that the temperature is increasing, demonstrating an exhaling pattern. The recorded results in Figure 21a indicate that the patient has an estimated breathing frequency of 0.48 Hz. On the other hand in Figure 21b, the estimated breathing frequency is around 0.22 Hz, corresponding to a relatively low breathing frequency, which could be the case of a patient quietly breathing, known as “Eupnea”. These results are reinforced by the study presented in Reference [40].

The breathing frequency of a healthy subject has been measured at multiple time intervals of one minute each, at the following scenarios: lying down flat on a bed with no movements (Scenario 1), walking normally in straight direction (Scenario 2), and walking fast in straight direction (Scenario 3). Table 1 presents the average of the measured breathing frequencies and the standard deviation, for each scenario.

The results in Table 1 show that the designed system is capable of determining the breathing frequency at different scenarios with minimum errors. It can also be observed that the measurement error increases as the breathing frequency increases. This error could be a consequence of the non-consistency of respiratory rate when someone is moving or walking fast.

## 6. Conclusions

We demonstrate the operation of a micro-fabricated RTD based breathing monitoring sensor. The optimized device is capable of transducing a change in air temperature while inhaling and exhaling into an output voltage variation used to determine the breathing frequency. Simulation results show that gold has the best performance among tested materials. The next steps of this work include optimizing the PCB design to reduce the final output signal noise, and testing the system with patients having different levels of breathing issues.

## Figures and Tables

**Table 1:** Average breathing frequency of a healthy subject at different scenarios.

	Average Breathing Frequency (Hz)	Standard Deviation (Hz)
**Scenario 1:** Healthy subject, lying down
flat on a bed, with no movements	0.28437	0.008615
**Scenario 2:** Healthy subject,
walking normally in straight direction	0.478028	0.020604
**Scenario 3:** Healthy subject,
walking fast in a straight direction	0.562761	0.026756

## Data Availability

Not applicable.
